# Enhanced extracellular raw starch-degrading α-amylase production in *Bacillus subtilis* by promoter engineering and translation initiation efficiency optimization

**DOI:** 10.1186/s12934-022-01855-9

**Published:** 2022-06-27

**Authors:** He Li, Dongbang Yao, Yan Pan, Xin Chen, Zemin Fang, Yazhong Xiao

**Affiliations:** 1 School of Life Sciences, Anhui University, Hefei, 230601 Anhui People’s Republic of China; 2 Anhui Key Laboratory of Modern Biomanufacturing, Hefei, 230601 Anhui People’s Republic of China

**Keywords:** Raw starch-degrading α-amylase, *Bacillus subtilis*, Dual-promoter, Translation initiation efficiency, 3-L Fermenter

## Abstract

**Background:**

A raw starch-degrading α-amylase from *Pontibacillus* sp. ZY (AmyZ1), previously screened by our laboratory, showed a promising application potential for starch-processing industries. However, the AmyZ1 secretory production still under investigation, which seriously restricts its application in the starch-processing industry. On the other hand, *Bacillus subtilis* is widely used to achieve the extracellular expression of target proteins.

**Results:**

AmyZ1 secretory production was achieved in *B. subtilis* and was enhanced by promoter engineering and translation initiation efficiency optimization. First, based on the different phase-dependent promoters, the dual-promoter P_*spoVG*_–P_*spoVG142*_ was constructed by combining dual-promoter engineering and promoter modification. The corresponding strain BZd34 showed an extracellular AmyZ1 activity of 1437.6 U/mL during shake flask cultivation, which was 3.11-fold higher than that of the original strain BZ1 (P_*groE*_). Then, based on translation initiation efficiency optimization, the best strain BZd343 containing optimized 5'-proximal coding sequence (opt3) produced the highest extracellular α-amylase activity of 1691.1 U/mL, which was 3.65-fold higher than that of the strain BZ1. Finally, cultivation of BZd343 in 3-L fermenter exhibited an extracellular AmyZ1 activity of 14,012 U/mL at 48 h, with productivity of 291.9 U/mL·h.

**Conclusions:**

This is the first report of recombinant expression of AmyZ1 in *B. subtilis* and the expression level of AmyZ1 represents the highest raw starch-degrading α-amylase level in *B. subtilis* to date. The high-level expression of AmyZ1 in this work provides a foundation for its industrial production. The strategies used in this study also provide a strategic reference for improving the secretory expression of other enzymes in *B. subtilis*.

**Supplementary Information:**

The online version contains supplementary material available at 10.1186/s12934-022-01855-9.

## Background

α-Amylase (EC3.2.1.1) is the enzyme that catalyze the hydrolysis of α-1,4-glucosidic bonds in starch, glycogen, and related polysaccharides in a random manner while releasing reducing groups [1]. It belongs to the family of GH13, GH57, GH119, and GH126 and has been widely applied in food, fermentation, textile, and papermaking industries [2, 3]. Starch is a critical natural raw material for the industrial production of bioethanol, organic acids, amino acids, and other products. Liquefaction with α-amylase is an essential step in starch-processing [4]. Traditional starch liquefaction is carried out by thermostable α-amylase at a high temperature of 95–105 °C [5]. However, this method is not conducive to saving production costs because it consumes much energy. By contrast, directly hydrolyzing the raw starch using raw starch-degrading α-amylase (RSDA) below the gelatinization temperature (40 °C) generates more interest [6]. For example, using RSDA in a low temperature to digest raw starch could bring about 10–20% reduction in energy consumption compared to the traditional starch conversion processes [5].

Thus far, although many RSDAs have been identified, little of them exhibit high specific activity toward raw starches [7]. In addition, the production of RSDAs with high substrate affinities and high specific activities toward raw starches remains a great challenge [6]. In our previous study, we reported a novel RSDA from *Pontibacillus* sp. ZY (named AmyZ1) with broad substrate specificity towards different raw starches at low temperatures (about 35 °C) and higher specific activity than other reported RSDAs [8]. Such properties make AmyZ1 an ideal candidate for the raw starch industry. However, when using *Escherichia coli* as a recombination production host, AmyZ1 primarily presents as insoluble inclusion bodies, thus hampers its industrial application. Therefore, choosing a suitable expression host for AmyZ1 soluble extracellular production is beneficial to promote its industrial application.

Compared to *E. coli*, *Bacillus subtilis* is a Gram-positive model bacterium with the advantages of strong protein secretion ability, easy to achieve soluble extracellular expression of proteins, and non-pathogenicity [9]. Therefore, *B. subtilis* has been an attractive strain for various industrial enzyme production [10]. However, there are still factors that limit the high-level expression of the target proteins in *B*. *subtilis*, such as secretion of multiple proteases, lack of strong promoter and efficient signal peptide (SP), and inefficient translation [11–13]. Many strategies have been developed to improve the recombinant expression level of target proteins in *B. subtilis*, such as host strain optimization [14], promoter and signal peptide optimization [15], and translation initiation efficiency optimization [16].

As a transcription-related regulatory factor, the promoter plays an important role in protein production [17]. Currently, promoters used in *B. subtilis* mainly include constitutive promoters and inducible promoters. Compared with constitutive promoters, inducible promoters have less influence on the growth of strains because they can control the expression cycle of target proteins. In past decades, many kinds of inducible promoter systems have been explored, such as tightly inducible expression systems induced by chemicals [18] and auto-inducible expression systems [19]. Compared with the chemical-dependent systems, auto-inducible phase-dependent expression systems without supernumerary inducers attracted much more attention because of their practical applications in the flexible and dynamic regulation of pathway genes [20]. In previous work, endogenous phase-dependent promoters of *B. subtilis* were divided into four classes (class I: exponential phase; class II: middle-log and early stationary phases; class III: lag-log and stationary phases; class IV: stationary phase) [21]. To date, auto-inducible phase-dependent promoters have also been successfully applied to high-level extracellular expression of the target proteins in *B. subtilis*. For example, the extracellular amidase activities mediated by phase-dependent promoters P_*lytR*_ (class III), P_*spoVG*_ (class II), P_*aprE*_ (class I), P_*yvyD*_ (class II), P_*ftsH*_ (class II), and P_*amyE*_ (class III) were 1.22-, 1.41-, 1.39-, 1.38-, 1.29-, and 1.46-fold higher than that mediated by widely used constitutive promoter P_*43*_, respectively [22].

Current research shows that constructing tandem promoters based on single promoters is an effective method to obtain novel high-efficiency strong promoters suitable for improving the extracellular expression of target proteins in *B. subtilis* [22]. The efficient extracellular expression of many target proteins is achieved in *B. subtilis* using the dual-promoter systems [17, 23]. For example, using the optimized dual-promoter P_*Bsamy*_-P_*Baamy*_, the alkaline serine protease (BcaPRO) activity in *B. subtilis* was 2.1- and 1.5-fold higher than those of strains with the promoters P_*Bsamy*_ and P_*Baamy*_, respectively [17]. Likewise, the aminopeptidase (AP) activity mediated by dual-promoter P_*HpaII*_-P_*gsiB*_ was 2.3- and 2.2-fold higher than those of the promoters P_*HpaII*_ and P_*gsiB*_, respectively [24].

In addition to the existing promoters, mutation of the promoter core regions such as − 35, − 16, − 10, and + 1 regions are also a practical approach to obtain the novel efficient promoters [25]. Currently, there are many reports on improving the extracellular expression level of the target proteins in *B. subtilis* by mutating the promoter core regions [26]. For example, the extracellular caseinolytic activity mediated by P_*2069M*_, obtained by improving the − 35 and − 10 regions of the promoter P_*2069*_, was twofold higher than that mediated by the promoter P_*2069*_ [26]. The β-galactosidase activity mediated by promoter P_*P3510*_, obtained by changing the − 35 and − 10 regions of the promoter P_*ylb*_, was ninefold higher than that mediated by the promoter P_*ylb*_ [25].

Apart from optimizing the transcription level of target genes, improving its translation efficiency is also an effective strategy to enhance the extracellular expression of target proteins in *B. subtilis* [27]. Many studies have shown that the secondary structures of mRNA present at the translation initiation region, including 5′-untranslated region and 5′-proximal coding sequence, greatly impact on the gene translation initiation efficiency [28]. Previous studies have reported that the recombinant expression level of the target proteins in *B. subtilis* can be improved by optimizing the translation initiation efficiency [13, 29]. For example, when a mutation occurred at the 5′-untranslated region of mRNA, the extracellular diacetylchitobiose deacetylase activity of the corresponding strain was 82-fold higher than that of the original strain [29]. Furthermore, the protein expression level of the best strain obtained by substituting synonymous codons at 5′-proximal coding sequence of mRNA was 530-fold higher than that of the original strain [30]. Nowadays, the tools like the ViennaRNA Web Services, UTR Designer, and RBS calculator are developed to predict mRNA secondary structures. Therefore, optimizing the mRNA secondary structure of the translation initiation region by software may be an effective means to improve the target protein secretory expression in *B. subtilis* [31].

**Fig. 1:**
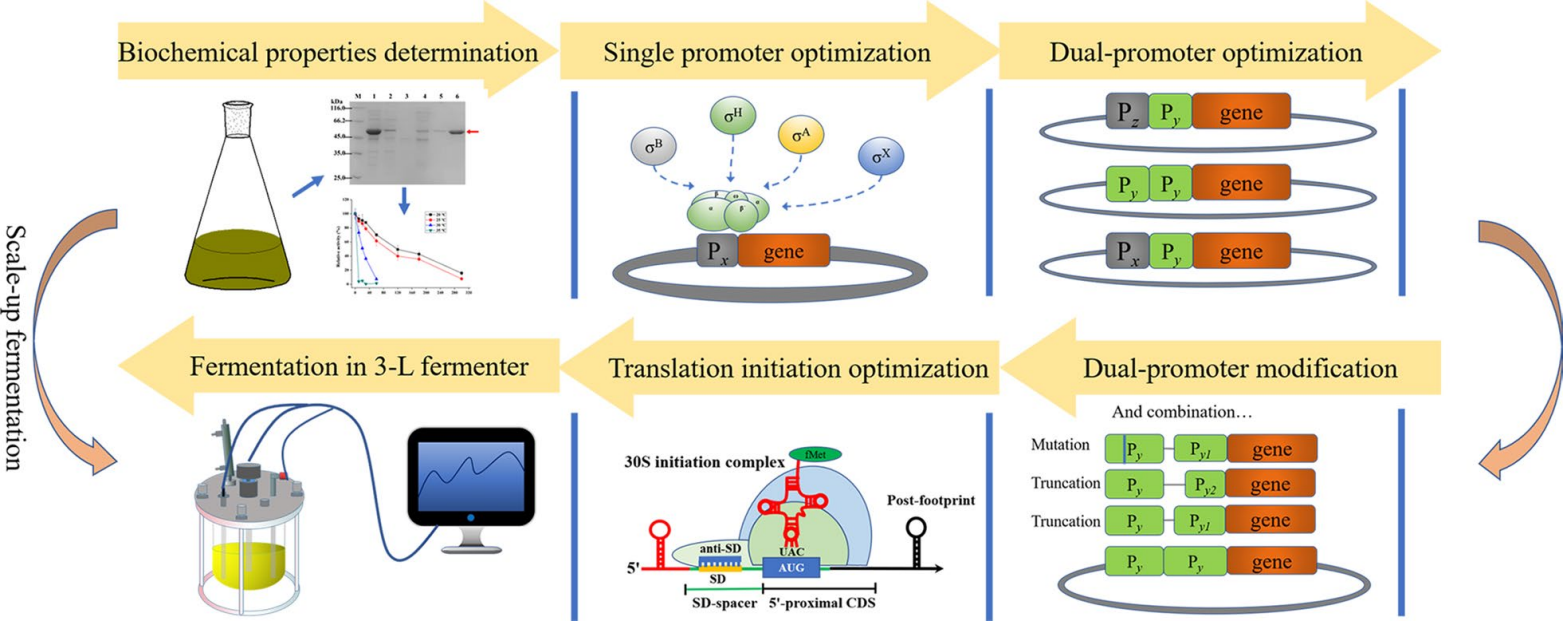
Sketch map of the strategies for enhancing the AmyZ1 production

In this study, AmyZ1 was first expressed extracellularly in *B. subtilis*. Then, AmyZ1 production was enhanced by promoter engineering strategies, including screening phase-dependent promoters, constructing dual-promoters, and modifying promoters. Next, the 5′-proximal coding sequence predicated by UTR Designer was used to optimize translation initiation efficiency of the AmyZ1 coding gene. Finally, the resulting *B. subtilis* recombinant strain was cultured in 3-L fermenters to verify its ability to produce AmyZ1. Sketch map of the above strategies could see in Fig. 1.

## Results and discussion

### Biochemical characterization of AmyZ1 expressed by *B. subtilis* WB600

In our previous study, AmyZ1 expressed in recombinant *E. coli* existed in the form of inclusion bodies [8]. In order to avoid this situation, the expression strain BZ1 was constructed by transforming *B. subtilis* WB600 with the recombinant vector pBHE, which contained the gene *AmyZ1*. After 48 h of fermentation in shake flasks, the extracellular AmyZ1 activity of BZ1 was 462.9 U/mL. Then, the AmyZ1 was successfully purified using the Ni-NAT system from the culture supernatant, which was verified by SDS-PAGE (Additional file 1: Fig. S1).

The biochemical characterization of recombinant AmyZ1 produced by *E. coli* had been determined, and it exhibited “cold-active” and broad pH catalytic abilities, suitable for the application in the starch-processing industry [8]. To verify whether AmyZ1 expressed in *B. subtilis* retained these excellent characteristics expressed in *E. coli*, its biochemical properties of AmyZ1 expressed in *B. subtilis* were determined*.* The specific activity of AmyZ1 expressed in *B. subtilis* toward raw rice was 16,773 U/mg (Additional file 1: Table S1) which was 1.32-fold higher than that of AmyZ1 expressed in *E. coli* (12,621 U/mg) [8]. This difference may be due to the denaturation and renaturation process required to obtain active AmyZ1 using *E. coli* [8], which may have a certain impact on the AmyZ1 structure, but this process can be effectively avoided when AmyZ1 is soluble extracellular expressed in *B. subtilis*.

**Fig. 2:**
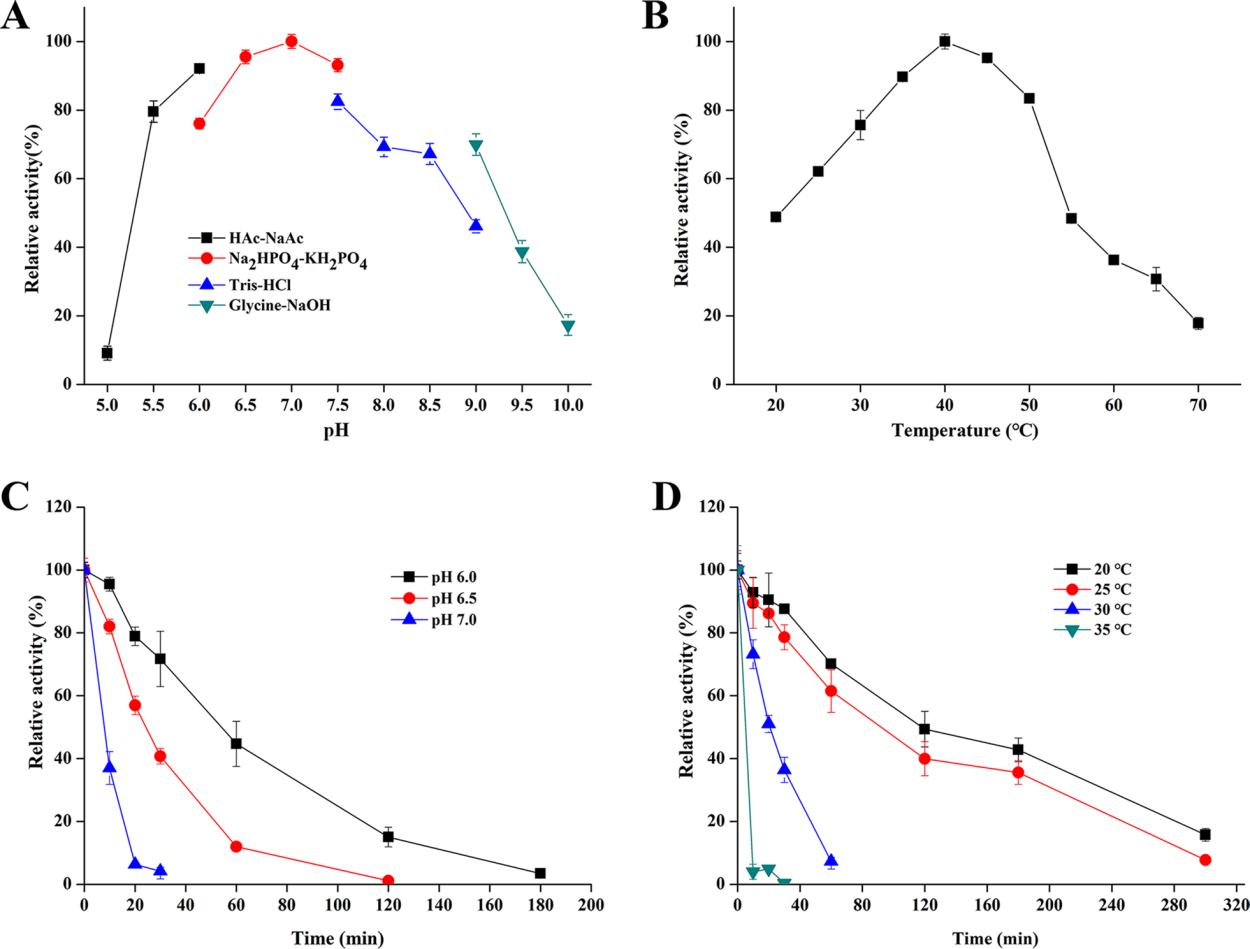
Effects of pH and temperature on AmyZ1 activity and stability. **A** The optimum pH. **B** The optimum temperature. **C** Effect of pH on enzyme stability. The purified enzyme was incubated in 50 mM Na_2_HPO_4_-KH_2_PO_4_ buffer (pH 6.0–7.0) at 30 °C. **D** Effect of temperature on enzyme stability. The purified enzyme was incubated in Na_2_HPO_4_-KH_2_PO_4_ buffer (50 mM, pH 6.5) at 20 to 35 °C. With raw rice starch as the substrate. Error bars represent standard deviation

In addition, the optimum pH and temperature of AmyZ1 produced by recombinant *B. subtilis* are 7.0 and 40 °C, respectively (Fig. 2A, B). Furthermore, the recombinant AmyZ1 maintained 80% and 75% activity at pH 5.5–7.5 and temperature 30–50 °C, respectively (Fig. 2A, B). In the absence of calcium ions, the half-life of the recombinant AmyZ1 produced by *B. subtilis* at pH 6.0 and temperature 20 °C was 50 and 120 min (Fig. 2C, D), respectively, which increased by about 35 and 105 min compared with those produced by *E. coli* under the same conditions, respectively [8].

In conclusion, the biochemical characteristics of recombinant AmyZ1 produced by *B. subtilis* were basically consistent with those produced by *E. coli*, even better in specific activity and low-temperature stability. These results suggested that AmyZ1 produced by *B. subtilis* is still suitable for application in the starch-processing industry.

### Promoter optimization for extracellular expression of AmyZ1 in *B. subtilis*

**Fig. 3:**
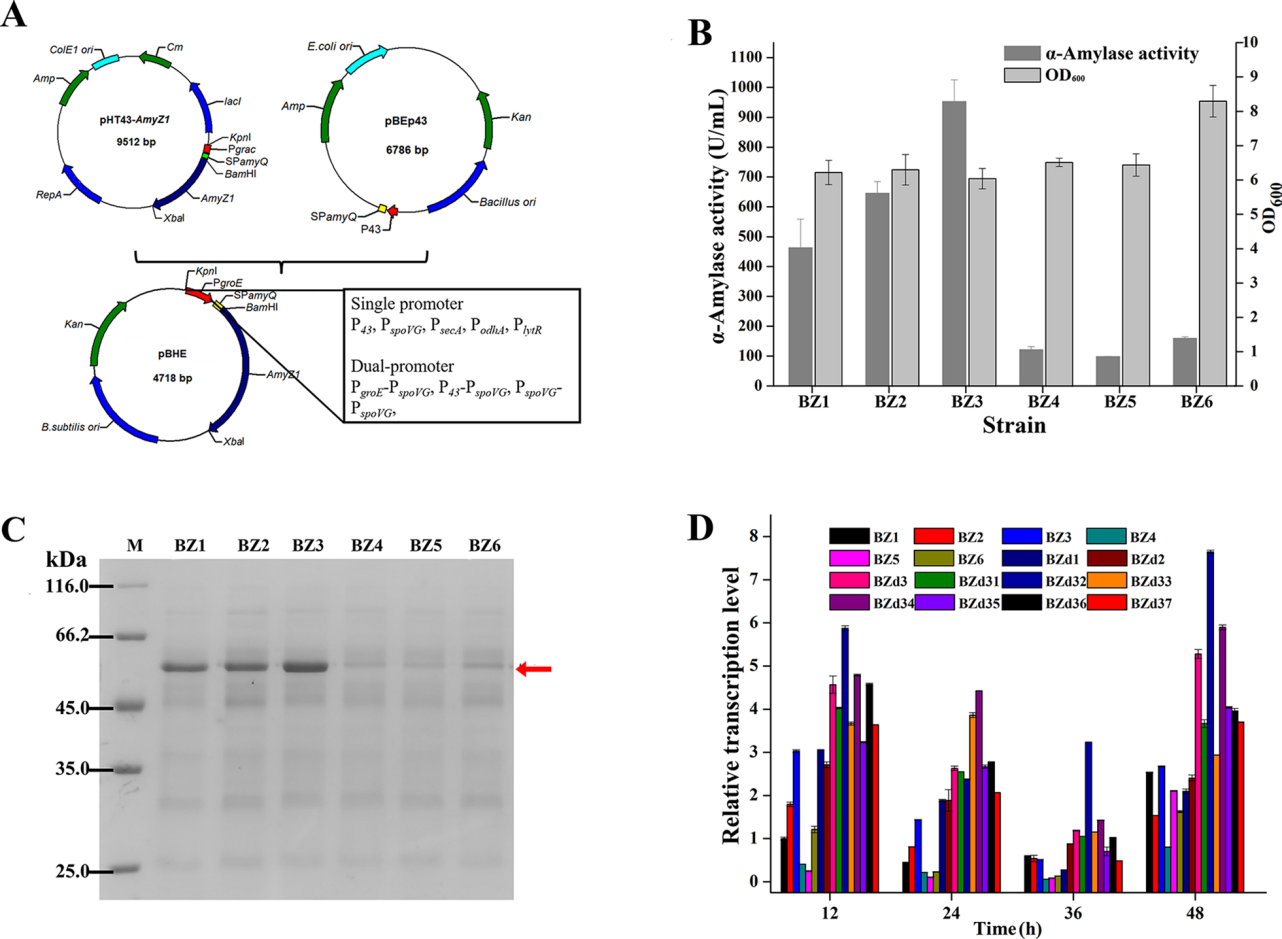
Effect of the single promoter optimization on the extracellular AmyZ1 expression. **A** Sketch of plamids construction. **B** The extracellular activities and cell densitities of strains with different single promoters. Error bars represent standard deviation. **C** SDS-PAGE analysis of AmyZ1. The target proteins were marked with arrow. **D** The AmyZ1 relative transcription level of all strains containing different promoters. Error bars represent standard deviation

The recombinant expression levels of AmyZ1 mediated by original promoter P_*groE*_ (σ^A^), the widely used constitutive promoter P_*43*_ (σ^AB^), and four endogenous phase-dependent promoters including P_*spoVG*_ (σ^H^, class II), P_*secA*_ (σ^A^, class III), P_*odhA*_ (σ^A^, class III), and P_*lytR*_ (σ^AX^, class III) in *B. subtilis* were investigated, respectively, to obtain an efficient promoter. The six plasmids containing different promoters pBHE (P_*groE*_), pBHP (P_*43*_), pBHS (P_*spoVG*_), pBHA (P_*secA*_), pBHO (P_*odhA*_), and pBHL (P_*lytR*_) were constructed (Fig. 3A) and transformed into *B. subtilis* WB600 to generate strains BZ1, BZ2, BZ3, BZ4, BZ5, and BZ6, respectively. After 48 h of fermentation in shake flasks, the extracellular AmyZ1 activities of strains BZ1 to BZ6 were 462.9, 701.8, 952.6, 114.5, 98.3, and 159.4 U/mL, respectively (Fig. 3B). To further verify the results shown above, the shake flask fermentation supernatant proteins of different recombinant strains were analyzed by SDS-PAGE (Fig. 3C). The thickness of the appropriate bands (around 55 kDa) was consistent with the AmyZ1 activities.

As shown in Fig. 3B, the extracellular AmyZ1 activity of strain BZ3 containing P_*spoVG*_ was the highest (952.6 U/mL), which was 2.06- and 1.36-fold higher than those of strains BZ1 and BZ2 containing P_*groE*_ (462.9 U/mL) and P_*43*_ (701.8 U/mL, p < 0.01), respectively. Similar to our results, the amidase activity and pullulanase activity mediated by promoter P_*spoVG*_ were 1.41- and 1.37-fold higher than those mediated by promoter P_*43*_, respectively [22, 32], suggesting that the phase-dependent promoter P_*spoVG*_ might be a strong promoter that could be used in other expression systems.

To investigate whether the extracellular AmyZ1 activities of different recombinant strains were related to the transcriptional intensity of their promoters, their *AmyZ1* mRNA levels were determined by quantitative real-time PCR. The *AmyZ1* mRNA levels of the strain BZ3 (P_*spoVG*_) were 3.03-, 3.17-, and 1.06-fold higher than those of strain BZ1 at 12, 24, and 48 h (p < 0.05), respectively (Fig. 3D). The *AmyZ1* mRNA levels of the strain BZ3 (P_*spoVG*_) were also 1.69-, 1.76-, and 1.74-fold higher than those of strain BZ2 containing widely used promoter P_*43*_ at 12, 24, and 48 h, respectively. These results were consistent with the corresponding AmyZ1 expression levels (Fig. 3B).

The AmyZ1 activities mediated by the promoters P_*secA*_, P_*odhA*_, and P_*lytR*_ were 114.5, 98.3, and 159.4 U/mL, respectively, lower than that mediated by promoter P_*spoVG*_ (952.6 U/mL) (Fig. 3B). However, this result was inconsistent with the previous work, where the four promoters mediated similar fluorescent intensities. The fluorescent intensities mediated by promoters P_*spoVG*_, P_*secA*_, P_*odhA*_, and P_*lytR*_ were about 28,000, 22,000, 23,000, and 30,000 units, respectively [21]. Indeed, almost no specific promoter is effective in various expression systems. This phenomenon may be caused by differences in the 5´-proximal coding sequence of the genes because their changes may affect the secondary structure of the mRNA, thus causing changes in translation initiation efficiency [33].

As for the reason why the *AmyZ1* transcription levels of all strains (BZ1 to BZ6) decreased first then increased (Fig. 3D), it suggested that some factors such as pH, temperature, and other factors may influence the transcription strength of the promoters [22, 34, 35]. Because the AmyZ1 activities of strains BZ1, BZ2, and BZ3 were higher than those of the other three strains BZ4, BZ5, and BZ6 (Fig. 3B), the corresponding promoters P_*groE*_, P_*43*_, and P_*spoVG*_ were employed in the subsequent experiments.

### Dual-promoter optimization for extracellular expression of AmyZ1 in *B. subtilis*

To explore the application potential of using dual-promoter engineering to improve the expression level of AmyZ1 in *B. subtilis*, several dual-promoter plasmids were constructed based on the single promoter optimization results. Since the single promoter adjacent to the target gene has a greater effect on the transcriptional activity of the dual-promoter [22], the promoters P_*groE*_, P_*43*_, and P_*spoVG*_ were fused to the 5´-end of promoter P_*spoVG*_, generating plasmids pBHES (P_*groE*_-P_*spoVG*_), pBHPS (P_*43*_-P_*spoVG*_), and pBHSS (P_*spoVG*_-P_*spoVG*_), respectively. These plasmids were then transformed into *B. subtilis* WB600, generating the strains BZd1, BZd2, and BZd3, respectively.

**Fig. 4:**
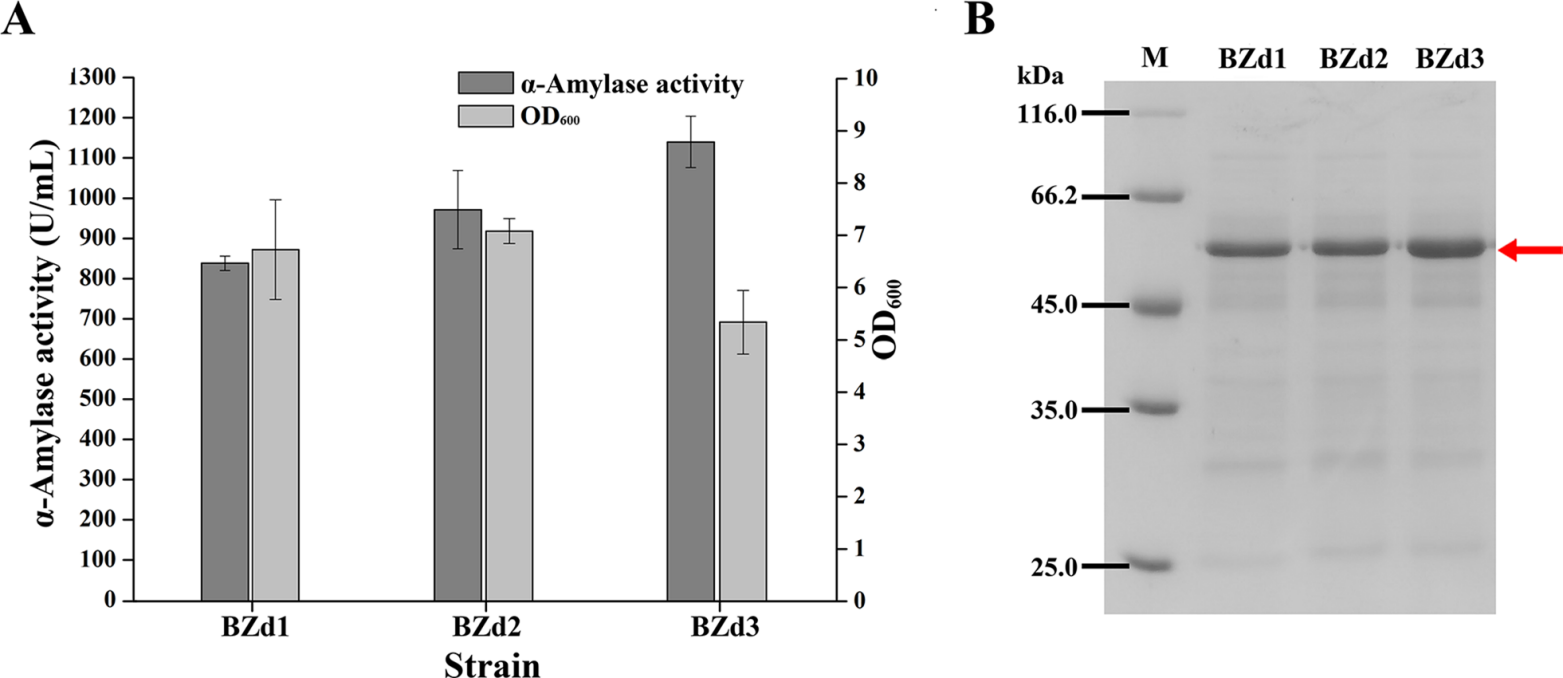
Shake flask culture of the recombinant strains mediated by the dual-promoters for AmyZ1 production. **A** The extracellular activities and cell densities of strains with different dual-promoters. Error bars represent standard deviation. **B** SDS-PAGE analysis of AmyZ1. The target proteins were marked with arrow

As shown in Fig. 4A, the extracellular AmyZ1 activities of strains BZd1, BZd2, and BZd3 were 838, 971.6, and 1139.8 U/mL after 48 h of fermentation in shake flasks, respectively. Among them, the strain BZd3 containing dual-promoter P_*spoVG*_-P_*spoVG*_ showed the highest activity, which was 1.19-fold higher than that of strain BZ3 (952.6 U/mL, p < 0.01) containing promoter P_*spoVG*_. SDS-PAGE analysis further confirmed the above results (Fig. 4B). Furthermore, the quantitative real-time PCR results of different recombinant strains showed that the *AmyZ1* mRNA level of the BZd3 strain containing dual-promoter P_*spoVG*_-P_*spoVG*_ was higher than those of other strains containing single promoters (BZ1 to BZ6) or dual-promoters (BZd1 and BZd2) during the whole fermentation stage (Fig. 3D). This may be why the extracellular AmyZ1 activity of strain BZd3 was higher than those of other strains (BZ1, BZ2, BZ3, BZ4, BZ5, BZ6, BZd1, and BZd2). In previous studies, it was reported that the extracellular amidase (*Bm*-Ami) activity mediated by the dual promoter P_*amyE*_-P_*43*_ (99.34 U/mL) was 1.8- and 2.63-fold higher than those mediated by promoters P_*amyE*_ (55.19 U/mL) and P_*43*_ (37.78 U/mL), respectively, and the *Bm*-*Ami* mRNA transcription level of the strain containing dual-promoter P_*amyE*_-P_*43*_ was higher than those of strains containing promoters P_*amyE*_ and P_*43*_ throughout the fermentation stage [22]. Therefore, it indicated that the higher enzyme activity mediated by dual-promoters may be due to the increase in its transcription level.

In this study, the extracellular AmyZ1 activities of strains BZd1 (P_*groE*_-P_*spoVG*_), BZd2 (P_*43*_-P_*spoVG*_), and BZd3 (P_*spoVG*_-P_*spoVG*_) containing dual-promoters were not the simple addition of the results seen with strains BZ1 (P_*groE*_), BZ2 (P_*43*_), and BZ3 (P_*spoVG*_) containing the corresponding single promoters. In addition, only dual-promoter P_*spoVG*_-P_*spoVG*_ mediated higher AmyZ1 activity than that mediated by P_*spoVG*_. In previous work, it was reported that only two of six dual-promoters mediated higher aminopeptidase activities than that mediated by the best single promoter and it indicated that the promoter origin influenced the cooperativity of the dual-promoter [24]. Besides that, another explanation for this result may be that the extracellular AmyZ1 activity is related to other factors, such as the translational efficiency of the mRNA.

Compared with the other strains containing single promoters, however, the biomass of strain BZd3 (P_*spoVG*_-P_*spoVG*_) decreased (Figs. 3B, 4A). This result is consistent with the previous finding that the maximum cell biomass of strains with dual-promoters (BL10/pP_*ylB*_-P_*43*_-GFP, BL10/pP_*gsiB*_-P_*43*_-GFP, and BL10/pP_*ykzA*_-P_*43*_-GFP) were all decreased [23]. Meanwhile, the fluorescence intensities produced by these strains containing dual-promoter were higher than that of strain with single promoter (BL10/pP43-GFP) and it was attributed to the increases in metabolic burdens in the strains with dual-promoters [23]. Therefore, the reduced biomass of strain BZd3 in this study may also be related to the increased metabolic burdens.

The three superior single promoters P_*groE*_, P_*43*_, and P_*spoVG*_ were inserted upstream of the best promoter P_*spoVG*_ to construct dual-promoters P_*groE*_-P_*spoVG*_, P_*43*_-P_*spoVG*_, and P_*spoVG*_-P_*spoVG*_. The AmyZ1 activities difference mediated by these dual-promoters was lower than that of the corresponding single promoters. For example, the AmyZ1 activities mediated by P_*groE*_ and P_*spoVG*_ were 462.9 and 952.6 U/mL, respectively, and the AmyZ1 activities mediated by P_*groE*_-P_*spoVG*_ and P_*spoVG*_-P_*spoVG*_ were 838 and 1139.8 U/mL, respectively. The enzyme activity mediated by P_*spoVG*_ is 2.06 times that mediated by P_*groE*_, while the enzyme activity mediated by P_*spoVG*_-P_*spoVG*_ is only 36% higher than that mediated by P_*groE*_-P_*spoVG*_. In previous works, a similar result was that the six dual-promoters with the same downstream promoter P_*amyQ'*_ also showed a weaker activity difference than that of single promoters [36]. And they attributed this result to the fact that the transcriptional strength of the dual-promoters was dominated by the promoter adjacent to the heterologous gene [36]. It may be the same phenomenon for the dual-promoters constructed in this study.

In addition, the promoter P_*spoVG*_ was identified as a class II promoter (middle-log and early stationary phases) with poor performance at stationary phase [21]. However, the *AmyZ1* mRNA level of the best dual-promoter P_*spoVG*_-P_*spoVG*_ at 48 h was highest throughout the fermentation stage (Fig. 3D). In previous study, the expression levels of aminopeptidase mediated by promoters P_*HpaII*_ and P_*gsiB*_ increased in the late exponential and stationary phases, respectively, however, the dual-promoter P_*HpaII*_–P_*gsiB*_ exhibited sharply increased expression at both the exponential and stationary phases, suggesting that the dual-promoter P_*HpaII*_–P_*gsiB*_ might possess a novel property of the promoter combination [24]. Therefore, based on the above results, we speculated that the dual-promoter P_*spoVG*_-P_*spoVG*_ constructed in this work may also possess a novel property of the promoter combination.

However, the extracellular AmyZ1 activity of strain BZd1 (838 U/mL) with dual-promoter P_*groE*_-P_*spoVG*_ was lower than that of strain BZ3 (952.6 U/mL) with the promoter P_*spoVG*_. What is more, the *AmyZ1* mRNA level of strain BZd1 was also lower than that of strain BZ3 at 36 and 48 h (p < 0.05) Similarly, the extracellular aminopeptidase (AP) activities of strains BSG21, BSG23, BSG25, and BSG26 containing the dual-promoters P_*HpaII*_-P_*HpaII*_, P_*HpaII*_-P_*43*_, P_*HpaII*_-P_*luxs*_, and P_*HpaII*_-P_*aprE*_ were 3.6, 5.1, 9.2, and 15.7 U/mL, respectively, lower than that of the strain BSG11 (31.9 U/mL) containing the single promoter P_*HpaII*_ [24]. These phenomena indicated that compared with the single promoters, the dual-promoters might not be practical for enhancing the extracellular production of the target proteins in *B. subtilis* under certain circumstances. It may be caused by factors such as the types of sigma factors and 5'-untranslation region sequences of genes [22, 29].

### Dual-promoter P_*spoVG*_-P_*spoVG*_ modification for extracellular expression of AmyZ1 in *B. subtilis*

**Fig. 5:**
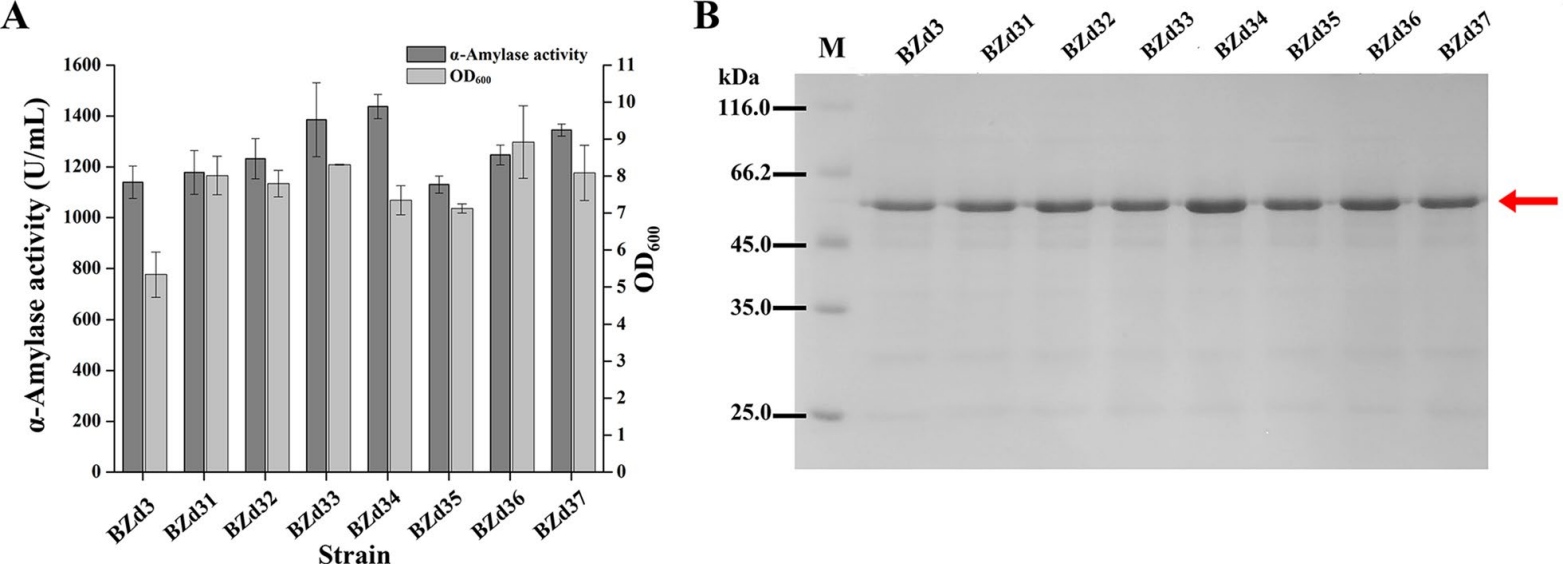
Effect of the dual-promoters modification on the extracellular AmyZ1 expression. **A** The extracellular activities and cell densities of strains with different modified dual-promoters. Error bars represent standard deviation. **B** SDS-PAGE analysis of AmyZ1. The target proteins were marked with arrow

Based on the results of Yang et al. [21], the promoter P_*spoVG*_ was selected to use in this study (approximately 300 bp). However, it was much longer than that reported by Zuber et al. [37] (the core region was approximately 90 bp). To ensure the peculiarity of phase-dependent promoter P_*spoVG*_, two truncated promoters P_*spoVG1*_ (120 bp) and P_*spoVG2*_ (180 bp) were cloned into plasmid pBHSS and resultant plasmids pBHSS1 (P_*spoVG*_-P_*spoVG1*_) and pBHSS2 (P_*spoVG*_-P_*spoVG2*_) were transformed further into *B. subtilis* WB600. The resulting recombinant strains BZd31 and BZd32 showed extracellular AmyZ1 activities of 1178.6 and 1232.3 U/mL (Fig. 5A), respectively, after 48 h of fermentation in shake flasks. Compared with the extracellular AmyZ1 activity of the strain BZd3 (1139.8 U/mL), although there was no significant change in strain BZd31 (p > 0.05), there had an 8% increase in strain BZd32 (p < 0.05). In addition, the *AmyZ1* mRNA levels of strain BZd32 were 1.29-, 2.72-, and 1.45-fold higher than those of strain BZd3 at 12 (p < 0.05), 36, and 48 h (p < 0.01), respectively (Fig. 3D). Therefore, this suggests that appropriate truncation of promoter upstream sequence may be an effective method to improve protein expression level because the promoter’s truncation may remove the response regions of some regulatory factors [38].

Since the single mutation C to T of the promoter P_*spoVG*_ at AT box can prevent the binding of AbrB repressor [39], we mutated P_*spoVG*_ to generate promoter P_*spoVG42*_. Then, the five plasmids pBHSS3 (P_*spoVG*_-P_*spoVG42*_), pBHSS4 (P_*spoVG*_-P_*spoVG142*_), pBHSS5 (P_*spoVG*_-P_*spoVG242*_), pBHSS6 (P_*spoVG42*_-P_*spoVG142*_), and pBHSS7 (P_*spoVG42*_-P_*spoVG242*_) were constructed based on P_*spoVG42*_, P_*spoVG1*_, and P_*spoVG2*_ and transformed into *B. subtilis* WB600 to obtain recombinant strains BZd33, BZd34, BZd35, BZd36, and BZd37, respectively. After 48 h of fermentation in shake flasks, the extracellular AmyZ1 activities of strains BZd33 to BZd37 were 1385, 1437.6, 1130.5, 1247.4, and 1344.6 U/mL, respectively (Fig. 5A). These results were consistent with the SDS-PAGE (Fig. 5B).

Notably, the strain BZd34 containing the promoter P_*spoVG*_-P_*spoVG142*_ exhibited the highest AmyZ1 activity, which was 1.25-fold higher than that of strain BZd31 (1178.6 U/mL, p < 0.01). In addition, the *AmyZ1* mRNA levels of strain BZd34 were 1.19-, 1.74-, 1.37-, and 1.62-fold higher than those of strain BZd31 at 12 (p < 0.001), 24, 36 (p < 0.001), and 48 h, respectively (Fig. 3D). These results suggested that the transcriptional strength of promoter P_*spoVG*_-P_*spoVG142*_ was enhanced by changing the core region of promoter P_*spoVG*_-P_*spoVG1*_, which might be an important reason why the extracellular AmyZ1 activity of strain BZd34 was higher than that of strain BZd31. This result was in accordance with previous finding that the β-galactosidase activity (about 7000 miller units) mediated by promoter P_*NBP3510*_, obtained by changing the UP element, − 35, and − 10 regions of the promoter P_*ylb*_, was 26-fold higher than that mediated by the promoter P_*ylb*_ and the highest transcription level of promoter P_*NBP3510*_ was 340-fold stronger than that of promoter P_*ylb*_ [25].

In the previous study, the strategies of dual-promoter construction included combining different single promoters, changing the position of a promoter and so on [22, 36]. In this study, a novel strategy that combined traditional dual-promoter construction with mutating the core region of the promoter was used to enhance the extracellular expression of AmyZ1 in *B. subtilis*. The resulting modified dual-promoters P_*spoVG*_-P_*spoVG2*_ (1232.3 U/mL), P_*spoVG*_-P_*spoVG42*_ (1385 U/mL), P_*spoVG*_-P_*spoVG142*_ (1437.6 U/mL), P_*spoVG42*_-P_*spoVG142*_ (1247.4 U/mL), and P_*spoVG42*_-P_*spoVG242*_ (1344.6 U/mL) mediated higher AmyZ1 activities than that mediated by original dual-promoter P_*spoVG*_-P_*spoVG*_ (1139.8 U/mL) (all p < 0.05), indicating that this method may be an effective strategy to improve the strength of dual-promoter.

### Optimization of translation initiation efficiency for extracellular expression of AmyZ1 in *B. subtilis*

**Fig. 6:**
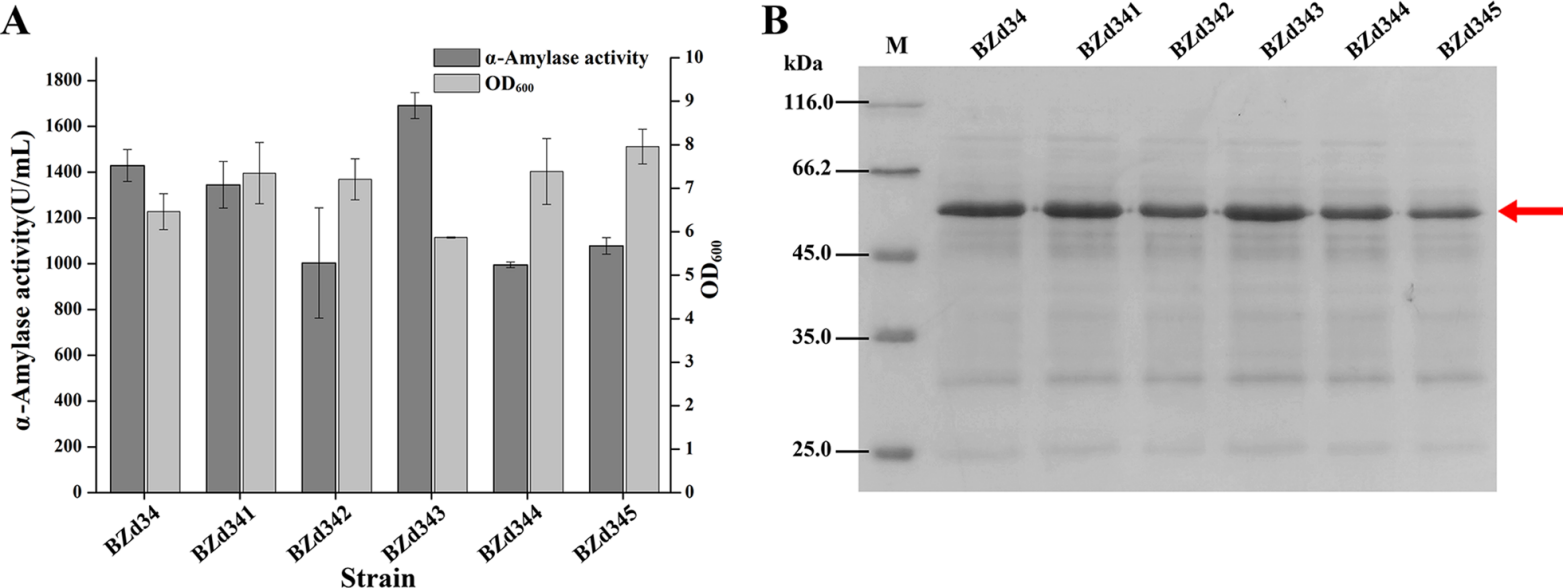
Effect of 5´-proximal coding sequence optimization on the extracellular AmyZ1 expression. **A** The extracellular activities and cell densities of strain with different optimization sequences. Error bars represent standard deviation. **B** SDS-PAGE analysis of AmyZ1. The target proteins were marked with arrow

As an important element of the gene 5′-end mRNA secondary structure, 5′-proximal coding sequences strongly affects gene translation efficiency [33]. Thus, it was an effective approach to optimize the translation efficiency by changing the 5′-proximal coding sequences [13]. To increase the free energy of *AmyZ1* mRNA secondary structure, the sequences of the first 11 codons of SP_*amyQ*_ were changed according to the prediction results of UTR Designer (see “Materials and methods”). The five plasmids pBHSS8, pBHSS9, pBHSS10, pBHSS11, and pBHSS12 containing the optimized 5'-proximal coding sequences (named opt1, opt2, opt3, opt4, and opt5, respectively) were transformed into *B. subtilis* WB600, generating strains BZd341, BZd342 BZd343, BZd344, and BZd345, respectively. The AmyZ1 activities of strains BZd341 to BZd345 were 1345, 1003.4, 1691.1, 995, and 1078.2 U/mL after 48 h of fermentation in shake flasks, respectively (Fig. 6A). SDS-PAGE analysis further confirmed this result (Fig. 6B). Among them, strain BZd343 exhibited the highest AmyZ1 activity, which was 1.18-fold higher than that of strain BZd34 (1437.6 U/mL, p < 0.01).

Based on the dual-promoter P_*spoVG*_-P_*spoVG142*_, only the optimized opt3 sequence had a positive effect on AmyZ1 expression. However, when replacing dual-promoter P_*spoVG*_-P_*spoVG142*_ with the single promoter P_*spoVG*_, all the five optimization sequences (opt1, opt2, opt3, opt4, and opt5, respectively) positively affected AmyZ1 expression (Additional file 1: Fig. S2). The AmyZ1 activities of the strains containing the five optimized sequences were 1.28- to 1.42-fold higher than that of the strain containing the original sequence, respectively, under single promoter mediation (Additional file 1: Fig. S2). This suggested that the strategy of optimizing translation initiation efficiency used in our study may involve only one of several important steps in the complex protein biosynthesis processes or the prediction was not accurate enough to the dual-promoter system because the prediction only contains about 25 bp upstream of the start codon.

In previous works, many researchers have tried to improve the RSDA production in different expression hosts using various strategies [40, 41]. For example, the recombinant RSDA activity produced by *Laceyella sacchari* LP175 was increased to 181.1 U/mL by medium optimization [42]. The AmyZ3 activity produced by *Bacillus amyloliquefaciens* BAZ3-16 with the in vitro methylation was 288.70 U/mL [41]. Using *E. coli* BL21 as the host, the recombinant Blamy-I activity reached 409.5 U/mL [43], which was the highest RSDA activity in shake flasks fermentation reported in the current literature as far as we know. In comparison, the RSDA activity of strain BZd343 was 1691.1 U/mL, which was 4.12-fold higher than that of the recombinant strain *E. coli* BL21 [43].

### Production of AmyZ1 in 3-L fermenter

**Fig. 7:**
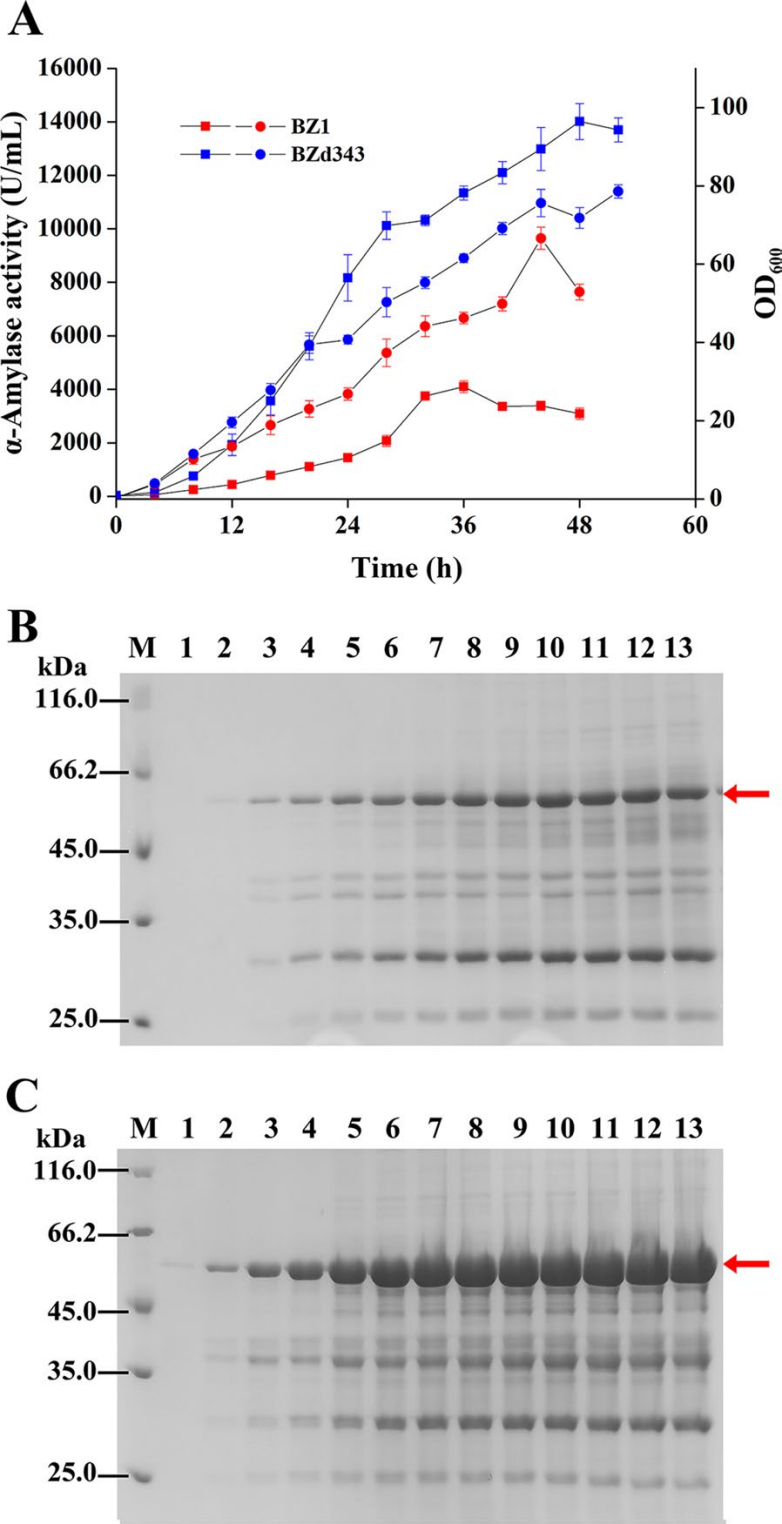
Scale-up (3-L) fermentation of the recombinant strains for AmyZ1 production. **A** The AmyZ1 activities and cell density at different times of strain BZ1 and BZd343. The squares represent AmyZ1 activity, and the circles represent cell density. Error bars represent standard deviation. **B**, **C** SDS-PAGE analysis of AmyZ1 in the supernatant at different time of strains BZ1 and BZd343, respectively. The target proteins were marked with arrow

To investigate the effects of promoter engineering and translation initiation efficiency optimization on the AmyZ1 extracellular expression in *B. subtilis*, the recombinant strains BZ1 (original strain) and BZd343 (optimized strain) were cultured in 3-L fermenters, respectively. The fed-batch strategy was used to obtain high cell density and high AmyZ1 activity in this study. During the fermentation process, the AmyZ1 activity in fermentation supernatants of strain BZ1 reached the maximum of 4097.8 U/mL at 36 h with productivity of 113.8 U/mL·h (Fig. 7A). The AmyZ1 activity of strain BZd343 reached the maximum of 14,012 U/mL at 48 h with productivity of 291.9 U/mL·h (Fig. 7A), which were 3.42- and 2.57-fold higher than those of strain BZ1, respectively. SDS-PAGE analysis further confirmed those results (Fig. 7B, C).

In a previous study, Tang et al. [41] reported the highest RSDA activity produced by the strain *B*. *amyloliquefaciens* BAZ3-16 in 5-L fermenter, which was 386.03 U/mL with productivity of 6.43 U/mL·h. In this study, the RSDA activity (14,012 U/mL) and productivity (291.9 U/mL·h) of strain BZd343 in 3-L fermenter were 36- and 45.4-fold higher than those of strain *B. amyloliquefaciens* BAZ3-16 [41], respectively. Although this is the highest recombinant expression level that has been reported for RSDA in *B. subtilis*, in order to meet the requirements of industrial applications better, follow-up strategies such as feed medium component or feed process optimization can be used to improve further the ability of the strain BZd343 to produce AmyZ1.

## Conclusions

The strategies of promoter engineering and translation initiation efficiency optimization successfully enhanced the secretory expression of AmyZ1 in *B. subtilis*. In this study, six single promoters were chose to evaluated their performances on AmyZ1 expression in shake-flask. The promoter P_*spoVG*_ mediated the highest AmyZ1 activity (952.6 U/mL). Three dual-promoters were constructed and screened under identical conditions. The dual-promoter P_*spoVG*_-P_*spoVG*_ showed the highest extracellular AmyZ1 activity (1139.8 U/mL). The modified promoter P_*spoVG*_-P_*spoVG142*_ further increased the AmyZ1 expression to 1437.6 U/mL. The AmyZ1 activities of the final recombinant strain BZd343 obtained by optimizing the 5'-proximal coding sequence were 1691.1 U/mL in shake flask culture and 14,012 U/mL in 3-L fermenter, respectively. It was the highest level of AmyZ1 secretory production in *B. subtilis* reported to date*.* This study thus provides an expression system with a potential for large-scale production of AmyZ1. This strategy might also be valuable for enhancing the production of other extracellular target proteins in *B. subtilis*.

## Materials and methods

### Strains, plasmids, and growth conditions

All strains and plasmids used in this study are listed in Table 1. *E. coli* JM109 was used as a host strain for plasmids construction. *B. subtilis* WB600 was used as a host strain for AmyZ1 expression.

**Table 1:** Strains and plasmids used in this study

Strains or plasmids	Description	Reference
Strains		
* E. coli* JM109	Clone strain	Takara
* Pontibacillus sp.* ZY	Clone strain	Our laboratory
* B. subtilis* 168	*trpC2*, clone strain	Our laboratory
* B. subtilis* WB600	*B. subtilis* 168 derivate, deficient in *nprE*, *aprE*, *epr, bpr*, *mpr*, *nprB,* expression strain	Our laboratory
BZ1	*B. subtilis* WB600/pBHE	This work
BZ2	*B. subtilis* WB600/pBHP	This work
BZ3	*B. subtilis* WB600/pBHS	This work
BZ4	*B. subtilis* WB600/pBHA	This work
BZ5	*B. subtilis* WB600/pBHO	This work
BZ6	*B. subtilis* WB600/pBHL	This work
BZd1	*B. subtilis* WB600/pBHES	This work
BZd2	*B. subtilis* WB600/pBHPS	This work
BZd3	*B. subtilis* WB600/pBHSS	This work
BZd31	*B. subtilis* WB600/pBHSS1	This work
BZd32	*B. subtilis* WB600/pBHSS2	This work
BZd33	*B. subtilis* WB600/pBHSS3	This work
BZd34	*B. subtilis* WB600/pBHSS4	This work
BZd35	*B. subtilis* WB600/pBHSS5	This work
BZd36	*B. subtilis* WB600/pBHSS6	This work
BZd37	*B. subtilis* WB600/pBHSS7	This work
BZd341	*B. subtilis* WB600/pBHSS8	This work
BZd342	*B. subtilis* WB600/pBHSS9	This work
BZd343	*B. subtilis* WB600/pBHSS10	This work
BZd344	*B. subtilis* WB600/pBHSS11	This work
BZd345	*B. subtilis* WB600/pBHSS12	This work
Plasmids		
pHT43	Amp^r^ (*E. coli*), Cm^r^ (*B. subtilis*), P_*groE*_, SP_*amyQ*_	[44]
pBEP43	Amp^r^ (*E. coli*), Kan^r^ (*B. subtilis*), P_*43*_, SP_*amyQ*_	[45]
pBHE	pHT43 derivative, P_*groE*_, SP_*amyQ*_	This work
pBHP	pHT43 derivative, P_*43*_, SP_*amyQ*_	This work
pBHS	pHT43 derivative, P_*spoVG*_, SP_*amyQ*_	This work
pBHA	pHT43 derivative, P_*secA*_, SP_*amyQ*_	This work
pBHO	pHT43 derivative, P_*odhA*_, SP_*amyQ*_	This work
pBHL	pHT43 derivative, P_*lytR*_, SP_*amyQ*_	This work
pBHES	pHT43 derivative, P_*groE*_-P_*spoVG*_, SP_*amyQ*_	This work
pBHPS	pHT43 derivative, P_*43*_-P_*spoVG*_, SP_*amyQ*_	This work
pBHSS	pHT43 derivative, P_*spoVG*_-P_*spoVG*_, SP_*amyQ*_	This work
pBHSS1	pHT43 derivative, P_*spoVG*_-P_*spoVG1*_, SP_*amyQ*_	This work
pBHSS2	pHT43 derivative, P_*spoVG*_-P_*spoVG2*_, SP_*amyQ*_	This work
pBHSS3	pHT43 derivative, P_*spoVG*_-P_*spoVG42*_, SP_*amyQ*_	This work
pBHSS4	pHT43 derivative, P_*spoVG*_-P_*spoVG142*_, SP_*amyQ*_	This work
pBHSS5	pHT43 derivative, P_*spoVG*_-P_*spoVG242*_, SP_*amyQ*_	This work
pBHSS6	pHT43 derivative, P_*spoVG42*_-P_*spoVG142*_, SP_*amyQ*_	This work
pBHSS7	pHT43 derivative, P_*spoVG42*_-P_*spoVG242*_, SP_*amyQ*_	This work
pBHSS8	pHT43 derivative, P_*spoVG*_-P_*spoVG142*_, SP_*opt1*_	This work
pBHSS9	pHT43 derivative, P_*spoVG*_-P_*spoVG142*_, SP_*opt2*_	This work
pBHSS10	pHT43 derivative, P_*spoVG*_-P_*spoVG142*_, SP_*opt3*_	This work
pBHSS11	pHT43 derivative, P_*spoVG*_-P_*spoVG142*_, SP_*opt4*_	This work
pBHSS12	pHT43 derivative, P_*spoVG*_-P_*spoVG142*_, SP_*opt5*_	This work

Luria–Bertani (LB) medium (1% tryptone, 1% NaCl, and 0.5% yeast extract) was used for seed culture and 2 × YT medium (1.6% tryptone, 0.5% NaCl, and 1% yeast extract) was used for *B. subtilis* shake flask fermentation. The seed culture was obtained by inoculating 5 mL of LB medium with 10 μL of bacterial solution from a glycerol tube cryopreserved at − 80 °C and incubated at 37 °C and 200 rpm for 10 h. The seed cultures (2%, v/v) were then transferred to 250 mL shake flasks containing 100 mL 2 × YT medium. Next, these cultures were incubated at 30 °C and 200 rpm for 48 h. The medium used in 3-L fermenter was modified 2 × YT medium, which included 2.4% tryptone, 0.75% NaCl, 1.5% yeast extract, and 1% glycerol. The feeding solution consisted of 40% glycerol, 6% tryptone, and 2% yeast extract. The optical density of recombinant strain at 600 nm (OD_600_) was measured by spectrophotometer (7200, UNICO, China). 30 mg/L kanamycin or 100 mg/L ampicillin was added when needed and 10 mM CaCl_2_ was added for *B. subtilis* shake flask fermentation and 3-L fermenter fermentation.

### Construction of various plasmids

The primers used in this study are listed in Additional file 1: Table S2. All promoters used in this study are listed in Table 2. The sequences of all promoters used are listed in Additional file 1: Table S3. All the recombinant plasmids constructed in this work were confirmed by DNA sequencing (Sangon Biotech Co., Ltd. Shanghai, China).

**Table 2:** Properties of promoters used for AmyZ1 expression

Promoter	Origin	Properties
P_*groE*_	*B. subtilis*	Recognized by σ^A^ RNA polymerase
P_*43*_	*B. subtilis*	Recognized by σ^A^ and σ^B^ RNA polymerase
P_*spoVG*_	*B. subtilis* 168	Recognized by σ^H^ RNA polymerase, Middle-log and early stationary phases
P_*secA*_	*B. subtilis* 168	Recognized by σ^A^ RNA polymerase, Lag-log and stationary phases
P_*odhA*_	*B. subtilis* 168	Recognized by σ^A^ RNA polymerase, Lag-log and stationary phases
P_*lytR*_	*B. subtilis* 168	Recognized by σ^A^ and σ^X^ RNA polymerase, lag-log and stationary phases

The *B. subtilis* 168 and the *Pontibacillus* sp. ZY genomes were extracted using the rapid bacterial genomic DNA isolation kit (Sangon Biotech, Shanghai, China). The *Pontibacillus* sp. ZY genome was used as the template for cloning *AmyZ1* (GenBank accession No. MH325467.1) using polymerase chain reactions (PCR) with the primers P1/P2. After being digested with *Bam*H I and *Xba* I, the *AmyZ1* gene was then ligated into plasmid pHT43 [44] to generate plasmid pHT43-*AmyZ1*.

The plasmids containing different single promoters were constructed as follows. Firstly, the fragment of promoter P_*groE*_ was cloned from pHT43 using the primers P3/P4. The fragment of promoter P_*43*_ was cloned from pBEP43 [45] using the primers P5/P6. The fragments of promoters P_*spoVG*_, P_*secA*_, P_*odhA*_, and P_*lytR*_ were cloned from *B. subtilis* 168 genome using the primers P7/P8, P9/P10, P11/P12, and P13/P14, respectively. One fragment derived from pHT43-*AmyZ1* containing ribosome binding site, SP_*amyQ*_, and *AmyZ1*, was cloned using the primers P15/P16. Another fragment derived from pBEP43 containing *B. subtilis ori* and *kan* gene was cloned using the primers P17/P18. Then, these two fragments were ligated by overlap PCR to generate a backbone plasmid fragment. Finally, the various promoters and the backbone plasmid fragment were ligated using POE-PCR [46], generating plasmids pBHE (P_*groE*_), pBHP (P_*43*_), pBHS (P_*spoVG*_), pBHA (P_*secA*_), pBHO (P_*odhA*_), and pBHL (P_*lytR*_), respectively.

Based on corresponding templates, the upstream promoters P_*groE*_, P_*43*_, and P_*spoVG*_ were cloned using the primers P3/P19, P5/P20, and P21/P22, respectively. Then, these three promoters were combined with P_*spoVG*_ to generate dual-promoter P_*groE*_-P_*spoVG*_, P_*43*_-P_*spoVG*_, and P_*spoVG*_-P_*spoVG*_, respectively. Finally, using the methods mentioned above, the three dual-promoters were ligated with backbone plasmid fragment to generate plasmids pBHES (P_*groE*_-P_*spoVG*_), pBHPS (P_*43*_-P_*spoVG*_), and pBHSS (P_*spoVG*_-P_*spoVG*_), respectively.

The modified dual-promoters were constructed based on the plasmid pBHSS, including truncation and mutation of promoter P_*spoVG*_. Two truncated promoters, P_*spoVG1*_ and P_*spoVG2*_, were cloned from *B. subtilis* 168 genome using primers P23/P25 and P24/P26, respectively. They deleted a 180 bp and 120 bp sequence compared with the original promoter P_*spoVG*_, respectively. These two promoters were then combined with promoter P_*spoVG*_ to generate plasmids pBHSS1 (P_*spoVG*_-P_*spoVG1*)_ and pBHSS2 (P_*spoVG*_-P_*spoVG2*_), respectively. The promoter P_*spoVG42*_ was obtained by overlapping the two fragments cloned from *B. subtilis* 168 genome using primers P21/P28 and P27/P6, respectively. The promoter P_*spoVG*_ was then combined with P_*spoVG42*_ to generate plasmid pBHSS3 (P_*spoVG*_-P_*spoVG42*_). Combining two strategies mentioned above, we constructed four plasmids containing different dual-promoters, named pBHSS4 (P_*spoVG*_-P_*spoVG142*_), pBHSS5 (P_*spoVG*_-P_*spoVG242*_), pBHSS6 (P_*spoVG42*_-P_*spoVG142*_), and pBHSS7 (P_*spoVG42*_-P_*spoVG242*_), respectively. All recombinant plasmids were transformed into *B. subtilis* WB600 following the method of Anagnostopoulos and Spizizen [47].

The optimization sequences of the 5′-proximal coding sequence of SP_*amyQ*_ (designated as opt1 to opt5) were amplified from plasmid pBHSS4 using P29/P16, P30/P16, P31/P16, P32/P16, and P33/P16 as primers, respectively. The backbone plasmid fragment was amplified from plasmid pBHSS4 using P17/P34 as primers. Then, this backbone plasmid fragment and five optimization sequences were combined by POE-PCR to generate plasmids pBHSS8 (opt1), pBHSS9 (opt2), pBHSS10 (opt3), pBHSS11 (opt4), and pBHSS12 (opt5), respectively.

### Analysis of AmyZ1 activity

The activity of AmyZ1 was determined using the dinitrosalicylic acid (DNS) assay method as described by Fang et al. [8], in which the reaction temperature was changed to 40 °C.

### Purification and biochemical properties determination of AmyZ1

The AmyZ1 was expressed in recombinant strain *B. subtilis* WB600 containing plasmid pBHE and then purified using the Ni-NAT system. The optimum temperature of AmyZ1 was determined at temperatures ranging from 20 to 70 °C. The optimum pH of AmyZ1 was assayed by measuring the activity in pH ranging from 5.0 to 10.0 at 40 °C using HAc-NaAc buffer (50 mM, pH 5.0–6.0), Na_2_HPO_4_-KH_2_PO_4_ buffer (50 mM, pH 6.0–7.5), Tris–HCl buffer (50 mM, pH 7.5–9.0), and Glycine–NaOH buffer (50 mM, pH 9.0–10), respectively. The highest activities of AmyZ1 were considered as 100% in the optimum temperature and optimum pH experiments, respectively. The thermostability of AmyZ1 was determined by incubating the AmyZ1 in Na_2_HPO_4_-KH_2_PO_4_ buffer (50 mM, pH 7.0) at temperatures ranging from 20 to 35 °C without Ca^2+^. The pH stability of AmyZ1 was determined by dispersing the AmyZ1 at pH 6.0, 6.5, and 7.0 without Ca^2+^, and the residual activities were measured at appropriate intervals. The initial activities of AmyZ1 were considered as 100% in the thermostability and pH stability experiments, respectively.

### Optimizing the translation initiation efficiency

Firstly, the 5'-proximal coding sequence of SP_*amyQ*_ was predicted by UTR Designer (https://sbi.postech.ac.kr/utr_designer/) [48] using its forward engineering mode to generate different optimization sequences. The 5'-end mRNA secondary structures of the best 24 optimization sequences were then calculated by the ViennaRNA Web Services (http://rna.tbi.univie.ac.at/cgi-bin/RNAWebSuite/RNAfold.cgi). The five optimized sequences (designated as opt1 to opt5) were finally obtained after removing the sequences containing the hairpin at the first 13 nucleotide residue of sequences (Table 3).

**Table 3:** Optimized 5′-proximal coding sequence used in this study

Nucleotide sequence	Free energy of second structure (kcal/mol)	Hairpin position	5′-Proximal coding sequence
ATGATTCAAAAACGAAAGCGGACAGTTTCGTTCAG	− 8.2	< 13	Control
ATGATACAAAAGAGGAAAAGGACGGTTAGTTTTAG	− 3.7	> 13	Opt1
ATGATACAAAAGCGAAAAAGGACGGTCAGTTTTAG	− 3.7	None	Opt2
ATGATACAAAAACGAAAGAGGACGGTCAGTTTTAG	− 3.8	> 13	Opt3
ATGATACAGAAACGAAAGCGGACGGTCAGTTTTAG	− 4.1	None	Opt4
ATGATACAAAAACGAAAGAGGACAGTCAGCTTTAG	− 4.2	> 13	Opt5

### Sodium dodecyl sulfate–polyacrylamide gel electrophoresis (SDS-PAGE)

The supernatant samples were mixed with 2 × loading buffer. The resulting mixtures were then heated at 100 °C for 10 min and evaluated with 12% polyacrylamide gel. The gel was stained with Coomassie Brilliant Blue R-250.

### Quantitative real-time PCR

Total RNAs of samples were isolated using the Bacteria Total RNA Isolation Kit (Sangon Biotech, Shanghai, China). Then, the cDNA was obtained using The Evo M-MLV RT Kit (AG, Hunan, China). The obtained cDNA was used as template for qPCR to measure the transcription level of gene *AmyZ1* with the 16S rRNA gene as the reference. The *AmyZ1* and 16S rRNA genes were amplified from the cDNA obtained above with primers P35/P36 and P37/P38, respectively. The reactions were carried out in the 96-well plates using SYBR Green Premix Pro Taq HS qPCR Kit (AG, Hunan, China) on a LightCycler 96 Real-Time PCR system (Roche, Basel, Switzerland) according to the manufacturer's protocols. The thermal cycling conditions contained an initial denaturation step (30 s at 95 °C); 50 cycles of denaturation (5 s at 95 °C); primer annealing and elongation (30 s at 60 °C); and a melt-curve step (0.3 °C/s, from 60 to 95 °C). The data was analyzed using the 2^−ΔΔCt^ methodology [49].

### Fermentation in 3-L fermenters

The seed culture was grown in a 250 mL shake flask containing 60 mL 2 × YT medium supplemented with 30 mg/L kanamycin at 37 °C and 200 rpm for 12 h. 3-L Fermenter (Eppendorf BioFlo 115, New Brunswick Scientific Co., Edison, NJ, USA) containing 1.2 L fermentation medium was then inoculated with the seed culture (5%, v/v). The dissolved oxygen tension was controlled by automatically adjusting the speed of the stirrer and airflow rate (2.0–5.0 L/min). The temperature was kept at 30 °C, and the pH was controlled at 7.0 using NH_4_OH and HCl. After fermentation about 10 h, the initial glycerol was consumed. Then, the fed-batch cultivation started with a feeding speed of 0–18 g glycerol per hour to maintain a glycerol concentration of 0.2 to 1.0 g/L. Samples were withdrawn every 4 h and centrifuged at 12,000×*g* for 10 min at 4 °C to obtain the culture supernatants.

### Analytic methods

All experiments were conducted independently in triplicate. Data was presented as the average ± standard deviation. Statistical analysis was conducted using Student’s *t*-test and differences resulting in p < 0.05 was considered statistically significant.

## Supplementary Information

**Additional file 1: Fig. S1** SDS-PAGE analysis of AmyZ1 purified using Ni-NAT system. **Fig. S2** Effect of the 5'-proximal coding sequence optimization on the extracellular AmyZ1 expression. **Table S1** Purification of AmyZ1. **Table S2** Primers used in this study. **Table S3** The sequences of different promoters used in this study.

## Data Availability

All data generated or analyzed during this study are included in this published article and its Additional files.

## Abbreviations

POE-PCR: Prolonged overlap extension polymerase chain reaction
RBS: Ribosome binding site
RSDA: Raw starch-degrading α-amylase
SP: Signal peptide
UTR: Untranslated region
