# Supplementary material for: Enhanced extracellular raw starch-degrading α-amylase production in Bacillus subtilis by promoter engineering and translation initiation efficiency optimization
Source: Microb Cell Fact. 2022 Jun 27;21:127. doi: 10.1186/s12934-022-01855-9 (PMC9235159; doi:10.1186/s12934-022-01855-9)
Supplement: Supplementary file 1 — Additional file 1: Fig. S1 SDS-PAGE analysis of AmyZ1 purified using Ni-NAT system. Fig. S2 Effect of the 5'-proximal coding sequence optimization on the extracellular AmyZ1 expression. Table S1 Purification of AmyZ1. Table S2 Primers used in this study. Table S3 The sequences of different promoters used in this study. [file 12934_2022_1855_MOESM1_ESM.docx]

**Additional information**

**Enhanced extracellular raw starch-degrading α-amylase production in *Bacillus subtilis* by promoter engineering and translation initiation efficiency optimization**

He Li ^1,2†^, Dongbang Yao ^1,2†^, Yan Pan ^1,2^, Xin Chen ^1,2^, Yazhong Xiao ^1,2*^, Zemin Fang ^1,2*^

^1^ School of Life Sciences, Anhui University, Hefei 230601, Anhui, RP China

^2^ Anhui Key Laboratory of Modern Biomanufacturing, Hefei, 230601 Anhui, RP China

^*^Correspondence

Yazhong Xiao, E-mail address: yzxiao@ahu.edu.cn

Zemin Fang, E-mail address: zemin_fang@ahu.edu.cn

^†^He Li and Dongbang Yao contributed equally to this work

**
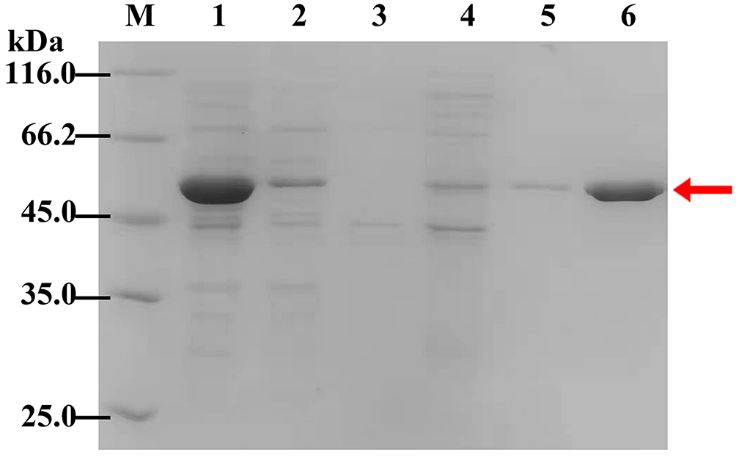
**

**Fig. S1** SDS-PAGE analysis of AmyZ1 purified using Ni-NAT system.

M: Marker. Lane 1: Crude enzyme. Lane 2: Sample of wash eluent. Lane 3-6: Samples of different eluents cotaining 5, 30, 60, and 200 mM imidazole, respectively. The target proteins were marked with arrow.

**
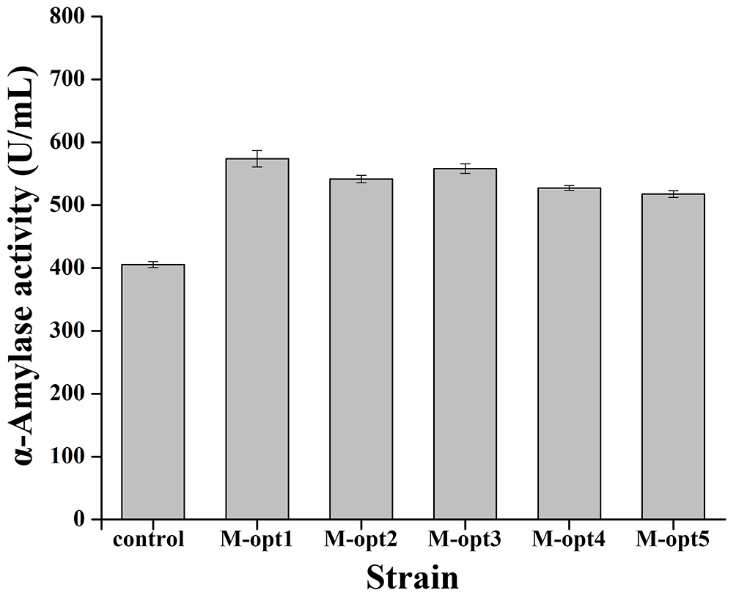
**

**Fig. S2** Effect of the 5'-proximal coding sequence optimization on the extracellular AmyZ1 expression. The control strain contained plasmid pHTS-*AmyZ1*, which replaced the promoter P*_groE_* with P*_spoVG_* on the basis of plasmid pHT43-*AmyZ1*. Based on the plasmid pHTS-*AmyZ1*, the five plasmids containing optimization sequences opt1, opt2, opt3, opt4 and opt5 were constructed and transformed into *B. subtilis* 168 to generate corresponding strains M-opt1, M-opt2, M-opt3, M-opt4, and M-opt5, respectively.

**Table S1** Purification of AmyZ1

| **Treatment** | **Volume (mL)** | **Total protein (mg)** | **Total activity (U)** | **Specific activity (U/mg)** | **Recovery (%)** | **Purification fold** |
| --- | --- | --- | --- | --- | --- | --- |
| Supernatant | 100 | 4.38 | 46290 | 10569 | 100 | 1 |
| Ni-NAT | 45 | 1.19 | 17083 | 14356 | 36.9 | 1.15 |
| Dialysis | 48 | 0.94 | 15767 | 16773 | 34.1 | 1.59 |

**Table S2** Primers used in this study

| **Primer** | **Sequence (5'-3')** |
| --- | --- |
| P1 | CGGGATCCATGGCAAGCAAGAATGGGAC |
| P2 | GCTCTAGATTACTTTTGTTTATATACCGAG |
| P3 | GAGGTTCGGATTCATCTATGGGTACCAGCTATTGTAAC |
| P4 | GATCCTTCCTCCTTTAATTGGGCCACAATTCTTATAATAAAG |
| P5 | GAGGTTCGGATTCATCTATGGGTACCTGCGGAAGTAAAC |
| P6 | GATCCTTCCTCCTTTAATTGGGCTGCAGCTATATAAAAGCATTAGTG |
| P7 | GTTCGGATTCATCTATGGGGGTACCGAGGAGAGCTTGGACATCGTC |
| P8 | GATCCTTCCTCCTTTAATTGGGCTGCAGCTAGTCCATGTATACCTCTC |
| P9 | GTTCGGATTCATCTATGGGGGTACCGAATAGAAGTGATAAATG |
| P10 | GATCCTTCCTCCTTTAATTGGGCTGCAGCAACGATATTTTACCACTG |
| P11 | GTTCGGATTCATCTATGGGGGTACCTGATAGGTGGTATGTTTTC |
| P12 | GATCCTTCCTCCTTTAATTGGGCTGCAGGCTATCACTTTATATTTTACATAATCG |
| P13 | GTTCGGATTCATCTATGGGGGTACCCTAACCCTACATAAGTACC |
| P14 | GATCCTTCCTCCTTTAATTGGGCTGCAGAAATTACTTTCATTATGAG |
| P15 | CCCAATTAAAGGAGGAAGGATC |
| P16 | CAACGCACCTTTCAGCCCTTCCACCCTTTCGATCAATTC |
| P17 | GAATTGATCGAAAGGGTGGAAGGGCTGAAAGGTGCGTTG |
| P18 | GTACCCCCATAGATGAATCCGAAC |
| P19 | CGTACACTTCGTTTACTTCCGCAAATTGTTATCCGCTCACAATTC |
| P20 | CGTACACTTCGTTTACTTCCGCAGCTATCACTTTATATTTTACATAATCG |
| P21 | GAGGTTCGGATTCATCTATG |
| P22 | CACTTCGTTTACTTCCGCACTATATAAAAGCATTAGTG |
| P23 | GATACACTAATGCTTTTATATAGCGAAATGAAAGCTTTATGA |
| P24 | GATACACTAATGCTTTTATATAGTGAAATAATAAGAAAAGTG |
| P25 | TCATAAAGCTTTCATTTCGCTATATAAAAGCATTAGTGTATC |
| P26 | CACTTTTCTTATTATTTCACTATATAAAAGCATTAGTGTATC |
| P27 | GTGTAACTATATCCTATTTTTTTAAAAAATATTTTAAAAACG |
| P28 | CGTTTTTAAAATATTTTTTAAAAAAATAGGATATAGTTACAC |
| P29 | CCAATTAAAGGAGGAAGGATCAATGATACAAAAGAGGAAAAGGACGGTTAGTTTTAG |
| P30 | CCAATTAAAGGAGGAAGGATCAATGATACAAAAGCGAAAAAGGACGGTCAGTTTTAG |
| P31 | CCAATTAAAGGAGGAAGGATCAATGATACAAAAACGAAAGAGGACGGTCAGTTTTAG |
| P32 | CCAATTAAAGGAGGAAGGATCAATGATACAGAAACGAAAGCGGACGGTCAGTTTTAG |
| P33 | CCAATTAAAGGAGGAAGGATCAATGATACAAAAACGAAAGAGGACAGTCAGCTTTAG |
| P34 | TGATCCTTCCTCCTTTAATTGG |
| P35 | GGCAGAGTGACAGGTGGT |
| P36 | GGCAACTAAGATCAAGGGTT |
| P37 | CGACGCAGTCAAGCACATAAAGC |
| P38 | AGTTCTCCCACATCGTTCTTCC |

**Table S3** The sequences of different promoters used in this study

| **Promoter** | **Sequence (5'-3')** |
| --- | --- |
| P*_groE_* | AGCTATTGTAACATAATCGGTACGGGGGTGAAAAAGCTAACGGAAAAGGGAGCGGAAAAGAATGATGTAAGCGTGAAAAATTTTTTATCTTATCACTTGAAATTGGAAGGGAGATTCTTTATTATAAGAATTGTGGAATTGTGAGCGGATAACAATT |
| P*_43_* | TGATAGGTGGTATGTTTTCGCTTGAACTTTTAAATACAGCCATTGAACATACGGTTGATTTAATAACTGACAAACATCACCCTCTTGCTAAAGCGGCCAAGGACGCTGCCGCCGGGGCTGTTTGCGTTTTTGCCGTGATTTCGTGTATCATTGGTTTACTTATTTTTTTGCCAAAGCTGTAATGGCTGAAAATTCTTACATTTATTTTACATTTTTAGAAATGGGCGTGAAGAAAAGCGCGCGATTATGTAAAATATAAAGTGATAGC |
| P*_spoVG_* | TGCGGAAGTAAACGAAGTGTACGGACAATATTTTGACACTCACAAACCGGCGAGATCTTGTGTTGAAGTCGCGAGACTCCCGAAGGATGCGTTAGTCGAGATCGAAGTTATTGCACTGGTGAAATAATAAGAAAAGTGATTCTGGGAGAGCCGGGATCACTTTTTTATTTACCTTATGCCCGAAATGAAAGCTTTATGACCTAATTGTGTAACTATATCCTATTTTTTCAAAAAATATTTTAAAAACGAGCAGGATTTCAGAAAAAATCGTGGAATTGATACACTAATGCTTTTATATAG |
| P*_odhA_* | GAATAGAAGTGATAAATGTTTTTCTCTCCAACTCAGAAATAGAAGAATCGCAAATGAAGACGATCCAGGATATGTACGGAAAATTCAGTATATTTGTCCCGGATGTTGATCAGCTACCGGATGTTTTGTATCCGCTGCTGAAAAAACTGCTTCATAAAAGCATAGGATAGCCCTTAATCCTATGCTTTTTGGCGTTTGTTTTTTCGAATGATTAAATTTTTTGTTTTTTATAAAGGTTTTTTACTATTTTGTGAACAATCAAGGTAGAATCAAATTGCAAACAGTGGTAAAATATCGTTG |
| P*_secA_* | CTAACCCTACATAAGTACCTTCTTTTGTTTCAATGTTACTGTCTGGCGATACATCTTCACCTTGACTCTTTTGACTATTAACCCCGCAACCCGAAAGAAGCAATATAAAGAACAGTAAAGCAATAAATTTTTTCATTTTTTTCACCTCATTATATTTTATCGTCAACCTATTTTATATTTTAAAGAAAAATTAAGAAACAATGAAACTTTTTTTTATAAAAAACGACTATTTTAGGATTTCATTCTTGTATTAAATAGAGTTGTATTTATTGGAAATTTAACTCATAATGAAAGTAATTT |
| P*_lytR_* | GAGGAGAGCTTGGACATCGTCCGTCAGAAACGCTTTAATTTAAAGCCGATGGATAGTGAAGAAGCGATCTTGCAAATGAATATGCTCGGCCATAATTTCTTTGTTTTCACAAATGCGGAAACAAACCTTACAAATGTCGTGTACCGCAGAAATGACGGGAAATATGGCTTAATTGAACCGACTGAATAATGAAGAGAAGCCTTCCGTGATGTCCGCGGAAGGTTTTTGTTTTTCTTATTTGCAAATTCTTTGGAAATAACAAAAGGTATGATATGATAATGAGAGGTATACATGGACTAG |
| P*_groE_*-P*_spoVG_* | AGCTATTGTAACATAATCGGTACGGGGGTGAAAAAGCTAACGGAAAAGGGAGCGGAAAAGAATGATGTAAGCGTGAAAAATTTTTTATCTTATCACTTGAAATTGGAAGGGAGATTCTTTATTATAAGAATTGTGGAATTGTGAGCGGATAACAATTtgcggaagtaaacgaagtgtacggacaatattttgacactcacaaaccggcgagatcttgtgttgaagtcgcgagactcccgaaggatgcgttagtcgagatcgaagttattgcactggtgaaataataagaaaagtgattctgggagagccgggatcacttttttatttaccttatgcccgaaatgaaagctttatgacctaattgtgtaactatatcctattttttcaaaaaatattttaaaaacgagcaggatttcagaaaaaatcgtggaattgatacactaatgcttttatatag |
| P*_43_*-P*_spoVG_* | TGATAGGTGGTATGTTTTCGCTTGAACTTTTAAATACAGCCATTGAACATACGGTTGATTTAATAACTGACAAACATCACCCTCTTGCTAAAGCGGCCAAGGACGCTGCCGCCGGGGCTGTTTGCGTTTTTGCCGTGATTTCGTGTATCATTGGTTTACTTATTTTTTTGCCAAAGCTGTAATGGCTGAAAATTCTTACATTTATTTTACATTTTTAGAAATGGGCGTGAAGAAAAGCGCGCGATTATGTAAAATATAAAGTGATAGCtgcggaagtaaacgaagtgtacggacaatattttgacactcacaaaccggcgagatcttgtgttgaagtcgcgagactcccgaaggatgcgttagtcgagatcgaagttattgcactggtgaaataataagaaaagtgattctgggagagccgggatcacttttttatttaccttatgcccgaaatgaaagctttatgacctaattgtgtaactatatcctattttttcaaaaaatattttaaaaacgagcaggatttcagaaaaaatcgtggaattgatacactaatgcttttatatag |
| P*_spoVG_*-P*_spoVG_* | TGCGGAAGTAAACGAAGTGTACGGACAATATTTTGACACTCACAAACCGGCGAGATCTTGTGTTGAAGTCGCGAGACTCCCGAAGGATGCGTTAGTCGAGATCGAAGTTATTGCACTGGTGAAATAATAAGAAAAGTGATTCTGGGAGAGCCGGGATCACTTTTTTATTTACCTTATGCCCGAAATGAAAGCTTTATGACCTAATTGTGTAACTATATCCTATTTTTTCAAAAAATATTTTAAAAACGAGCAGGATTTCAGAAAAAATCGTGGAATTGATACACTAATGCTTTTATATAGtgcggaagtaaacgaagtgtacggacaatattttgacactcacaaaccggcgagatcttgtgttgaagtcgcgagactcccgaaggatgcgttagtcgagatcgaagttattgcactggtgaaataataagaaaagtgattctgggagagccgggatcacttttttatttaccttatgcccgaaatgaaagctttatgacctaattgtgtaactatatcctattttttcaaaaaatattttaaaaacgagcaggatttcagaaaaaatcgtggaattgatacactaatgcttttatatag |
| P*_spoVG_*-P*_spoVG1_* | TGCGGAAGTAAACGAAGTGTACGGACAATATTTTGACACTCACAAACCGGCGAGATCTTGTGTTGAAGTCGCGAGACTCCCGAAGGATGCGTTAGTCGAGATCGAAGTTATTGCACTGGTGAAATAATAAGAAAAGTGATTCTGGGAGAGCCGGGATCACTTTTTTATTTACCTTATGCCCGAAATGAAAGCTTTATGACCTAATTGTGTAACTATATCCTATTTTTTCAAAAAATATTTTAAAAACGAGCAGGATTTCAGAAAAAATCGTGGAATTGATACACTAATGCTTTTATATAGcgaaatgaaagctttatgacctaattgtgtaactatatcctattttttcaaaaaatattttaaaaacgagcaggatttcagaaaaaatcgtggaattgatacactaatgcttttatatag |
| P*_spoVG_*-P*_spoVG2_* | TGCGGAAGTAAACGAAGTGTACGGACAATATTTTGACACTCACAAACCGGCGAGATCTTGTGTTGAAGTCGCGAGACTCCCGAAGGATGCGTTAGTCGAGATCGAAGTTATTGCACTGGTGAAATAATAAGAAAAGTGATTCTGGGAGAGCCGGGATCACTTTTTTATTTACCTTATGCCCGAAATGAAAGCTTTATGACCTAATTGTGTAACTATATCCTATTTTTTCAAAAAATATTTTAAAAACGAGCAGGATTTCAGAAAAAATCGTGGAATTGATACACTAATGCTTTTATATAGgaaataataagaaaagtgattctgggagagccgggatcacttttttatttaccttatgcccgaaatgaaagctttatgacctaattgtgtaactatatcctattttttcaaaaaatattttaaaaacgagcaggatttcagaaaaaatcgtggaattgatacactaatgcttttatatag |
| P*_spoVG_*-P*_spoVG42_* | TGCGGAAGTAAACGAAGTGTACGGACAATATTTTGACACTCACAAACCGGCGAGATCTTGTGTTGAAGTCGCGAGACTCCCGAAGGATGCGTTAGTCGAGATCGAAGTTATTGCACTGGTGAAATAATAAGAAAAGTGATTCTGGGAGAGCCGGGATCACTTTTTTATTTACCTTATGCCCGAAATGAAAGCTTTATGACCTAATTGTGTAACTATATCCTATTTTTTCAAAAAATATTTTAAAAACGAGCAGGATTTCAGAAAAAATCGTGGAATTGATACACTAATGCTTTTATATAGtgcggaagtaaacgaagtgtacggacaatattttgacactcacaaaccggcgagatcttgtgttgaagtcgcgagactcccgaaggatgcgttagtcgagatcgaagttattgcactggtgaaataataagaaaagtgattctgggagagccgggatcacttttttatttaccttatgcccgaaatgaaagctttatgacctaattgtgtaactatatcctatttttt**T**aaaaaatattttaaaaacgagcaggatttcagaaaaaatcgtggaattgatacactaatgcttttatatag |
| P*_spoVG_*-P*_spoVG142_* | TGCGGAAGTAAACGAAGTGTACGGACAATATTTTGACACTCACAAACCGGCGAGATCTTGTGTTGAAGTCGCGAGACTCCCGAAGGATGCGTTAGTCGAGATCGAAGTTATTGCACTGGTGAAATAATAAGAAAAGTGATTCTGGGAGAGCCGGGATCACTTTTTTATTTACCTTATGCCCGAAATGAAAGCTTTATGACCTAATTGTGTAACTATATCCTATTTTTTCAAAAAATATTTTAAAAACGAGCAGGATTTCAGAAAAAATCGTGGAATTGATACACTAATGCTTTTATATAGcgaaatgaaagctttatgacctaattgtgtaactatatcctatttttt**T**aaaaaatattttaaaaacgagcaggatttcagaaaaaatcgtggaattgatacactaatgcttttatatag |
| P*_spoVG_*-P*_spoVG242_* | TGCGGAAGTAAACGAAGTGTACGGACAATATTTTGACACTCACAAACCGGCGAGATCTTGTGTTGAAGTCGCGAGACTCCCGAAGGATGCGTTAGTCGAGATCGAAGTTATTGCACTGGTGAAATAATAAGAAAAGTGATTCTGGGAGAGCCGGGATCACTTTTTTATTTACCTTATGCCCGAAATGAAAGCTTTATGACCTAATTGTGTAACTATATCCTATTTTTTCAAAAAATATTTTAAAAACGAGCAGGATTTCAGAAAAAATCGTGGAATTGATACACTAATGCTTTTATATAGgaaataataagaaaagtgattctgggagagccgggatcacttttttatttaccttatgcccgaaatgaaagctttatgacctaattgtgtaactatatcctatttttt**T**aaaaaatattttaaaaacgagcaggatttcagaaaaaatcgtggaattgatacactaatgcttttatatag |
| P*_spoVG42_*-P*_spoVG142_* | TGCGGAAGTAAACGAAGTGTACGGACAATATTTTGACACTCACAAACCGGCGAGATCTTGTGTTGAAGTCGCGAGACTCCCGAAGGATGCGTTAGTCGAGATCGAAGTTATTGCACTGGTGAAATAATAAGAAAAGTGATTCTGGGAGAGCCGGGATCACTTTTTTATTTACCTTATGCCCGAAATGAAAGCTTTATGACCTAATTGTGTAACTATATCCTATTTTTT**T**AAAAAATATTTTAAAAACGAGCAGGATTTCAGAAAAAATCGTGGAATTGATACACTAATGCTTTTATATAGcgaaatgaaagctttatgacctaattgtgtaactatatcctatttttt**T**aaaaaatattttaaaaacgagcaggatttcagaaaaaatcgtggaattgatacactaatgcttttatatag |
| P*_spoVG42_*-P*_spoVG242_* | TGCGGAAGTAAACGAAGTGTACGGACAATATTTTGACACTCACAAACCGGCGAGATCTTGTGTTGAAGTCGCGAGACTCCCGAAGGATGCGTTAGTCGAGATCGAAGTTATTGCACTGGTGAAATAATAAGAAAAGTGATTCTGGGAGAGCCGGGATCACTTTTTTATTTACCTTATGCCCGAAATGAAAGCTTTATGACCTAATTGTGTAACTATATCCTATTTTTT**T**AAAAAATATTTTAAA |
|  | AACGAGCAGGATTTCAGAAAAAATCGTGGAATTGATACACTAATGCTTTTATATAGgaaataataagaaaagtgattctgggagagccgggatcacttttttatttaccttatgcccgaaatgaaagctttatgacctaattgtgtaactatatcctatttttt**T**aaaaaatattttaaaaacgagcaggatttcagaaaaaatcgtggaattgatacactaatgcttttatatag |

Mutation sites are shown in bold.
